# MMPs’ Impact on Carcinogenesis in Specific Organs and Systems

**DOI:** 10.3390/cancers18121900

**Published:** 2026-06-11

**Authors:** Marta Lewoc-Magnuszewska, Barbara Choromańska, Mateusz Maciejczyk, Jacek Dadan, Piotr Myśliwiec

**Affiliations:** 1 1st Department of General and Endocrine Surgery, Medical University of Bialystok, 24a M. Sklodowskiej-Curie Street, 15-276 Bialystok, Poland; barbara.choromanska@umb.edu.pl (B.C.); jacek.dadan@umb.edu.pl (J.D.); 2 Department of Hygiene, Epidemiology and Ergonomics, Medical University of Bialystok, 2c A. Mickiewicza, 15-369 Bialystok, Poland; mateusz.maciejczyk@umb.edu.pl; 3 Department of Minimally Invasive Surgery, Medical University of Bialystok, 24a M. Sklodowskiej-Curie Street, 15-276 Bialystok, Poland; piotr.mysliwiec@umb.edu.pl

**Keywords:** MMPs, metalloproteinases, carcinogenesis

## Abstract

Cancer remains a primary global health and economic challenge, characterized by complex mechanisms of carcinogenesis and metastasis. Central to these processes is the dysregulation of matrix metalloproteinases, a family of zinc-dependent proteases. While traditionally recognized for their role in extracellular matrix remodeling, MMPs are now understood as versatile regulators of both intra- and extracellular signaling. This review explores the characteristics and diverse functional roles of MMPs in representative cancers, highlighting their transition from simple matrix degraders to complex modulators of the tumor microenvironment. Current evidence indicates that non-selective inhibition of all MMPs is unlikely to be an effective therapeutic strategy. Instead, a more targeted approach focusing on specific MMPs and disease stages may be required.

## Simple Summary

Cancer remains a severe global health burden and a leading cause of premature death. While the exact mechanisms of cancer development and metastasis are not fully understood, recent research highlights that disruptions in matrix metalloproteinase (MMP) homeostasis play a critical role in its pathogenesis. MMPs facilitate cancer cell proliferation, tissue invasion, angiogenesis, and evasion of apoptosis. Across various malignancies (including breast, lung, gastrointestinal, genitourinary, skin, nervous, and endocrine cancers), elevated levels of specific MMPs consistently correlate with advanced tumor stages, metastasis, and poor prognosis. Consequently, specific MMPs show immense promise as targeted diagnostic biomarkers and therapeutic objectives. The aim of this review was to synthesize the current literature on the involvement of MMPs in carcinogenesis and metastasis in specific cancers. Ultimately, a targeted approach focusing on distinct MMPs and disease stages is essential to identifying reliable, cancer-specific diagnostic biomarkers and effective therapeutic objectives. This review presents current research on the involvement of specific MMPs at the developmental stages of common cancers, which may be useful for planning cancer-suppression studies.

## 1. Introduction

Cancer is a major social, public health, and economic problem, accounting for one-sixth of all deaths and nearly 30% of premature deaths from noncommunicable diseases worldwide. It is one of the three leading causes of death among adults under 70 years of age in 177 countries [1,2,3].

Despite extensive research efforts, the mechanisms underlying carcinogenesis and metastasis remain incompletely understood. Genetic and epigenetic alterations constitute the foundation of cancer development, with the accumulation of endogenous and exogenous factors considered a major driver of carcinogenesis [4]. Recent studies highlight that disturbances in matrix metalloproteinase (MMP) homeostasis play a crucial role in cancer pathogenesis [5,6,7,8,9,10,11,12].

MMPs belong to the metzincin family of proteases, comprising 28 members, of which 24 have been identified in humans [13]. Their name reflects key structural features, including a methionine residue at the active site and the requirement for zinc in catalytic activity [13]. The typical multidomain structure of MMPs includes an N-terminal signal peptide that directs secretion, a propeptide domain (~80 amino acids) that maintains the enzyme in a latent form, a catalytic domain (~170 amino acids) containing a conserved Zn^2+^-binding site, and a C-terminal hemopexin domain (~200 amino acids) responsible for substrate specificity [14,15].

MMPs are classified into subgroups based on sequence similarity, domain organization, and substrate specificity. These include collagenases (MMP-1, -8, -13), gelatinases (MMP-2, -9), stromelysins (MMP-3, -10, -11), matrilysins (MMP-7, -26), membrane-type MMPs (MMP-14 to -17, -24, -25), and others (MMP-12, -19 to -23, -27, -28) [13,16,17,18].

## 2. MMPs Roles

MMPs regulate the extracellular matrix (ECM) by degrading and remodeling its components, particularly collagens and proteoglycans. Although other proteases also contribute to ECM turnover, MMPs play a central role in this process [16,17,19].

Beyond ECM remodeling, MMPs are involved in numerous physiological processes, including embryonic development, branching morphogenesis, wound healing, and angiogenesis. They modulate growth factors and cytokines, including vascular endothelial growth factor (VEGF), thereby influencing both vasculogenesis and angiogenesis [16]. This classic role is characterized by the highest standard of validation. Extensive preclinical in vivo mouse models (knockout and transgenic lines) confirm that altering the balance between active MMPs and tissue inhibitors of MMPs (TIMPs) accelerates local tissue invasion, tumor vascular sprouting, and colonization of distant organs [9,20,21,22].

Importantly, MMPs have functions extending beyond ECM degradation. Approximately 31% of their substrates are ECM components, whereas the majority are non-ECM proteins [16,23]. Their actions encompass their classical roles in the ECM and newly discovered functions in intracellular signaling compartments. The primary structural role of MMPs involves the proteolytic degradation and remodeling of mechanical barriers in the tumor microenvironment [6]. The main substrates for these actions are structural collagens, proteoglycans, laminin, and fibronectin [6,13]. Collagenases (MMP-1, -8, -13) and gelatinases (MMP-2, -9) systematically degrade the physical constraints of the basement membrane and the surrounding interstitial stroma [6,8]. Membrane-type MMPs (MMP-14/MT1-MMP) act directly on the cell surface, clearing invasion pathways [24].

Consequently, MMPs are now recognized as key regulators of intra- and extracellular signaling pathways, modulating cellular communication through the cleavage of cytokines, chemokines, and growth factors [20,25].

Intracellular MMPs enter the cytosol, mitochondria, and nucleus via mechanisms that are still being explored [5,16,26]. In the cytosol and on the cell surface, MMPs process cytokines, chemokines, and non-ECM surface receptors [16,23]. For example, MMP-7 protects cancer cells from immune recognition and programmed cell death by systematically cleaving the surface Fas ligand (FasL) [27]. This cleavage disrupts key apoptotic cascades, halting further activation of caspase-8 and caspase-10 [28]. MMPs’ intracellular function and their mechanisms of internalization and trafficking are still incompletely understood, and they remain confirmed mainly at the in vitro level [3,6]. Most supporting evidence is based on cell culture assays, localized transfection models, and high-throughput terminator-tagged in vitro proteomics (such as TAILS) [16,20,24]. Translating these intracellular mechanisms into animal models is technically problematic. It remains difficult to separate the enzyme’s intracellular signaling effects from its classical extracellular tissue remodeling functions without disrupting overall systemic homeostasis [5,10].

MMPs also participate in inflammatory responses by regulating leukocyte migration, chemokine gradients, and cytokine activation [20,21].

This review summarizes the complex roles of MMPs in physiological processes and their involvement in cancer development and progression.

### MMP Activators and Inhibitors

MMP activity is regulated at multiple levels, including proteolytic activation, transcriptional control, inhibition by endogenous proteins, and redox-dependent mechanisms [17,24].

Activation of MMPs occurs through proteolytic cleavage of the N-terminal propeptide by other MMPs or serine proteases such as trypsin, plasmin, and leukocyte elastase [17,24]. Transcription of MMPs genes is tightly controlled by cytokines, growth factors, and chemokines, and varies depending on cell type, cellular stress, and specific transcription factors (for example, phorbol esters, integrin-derived signals, and extracellular matrix proteins) [16,29].

Reactive oxygen species (ROS) and reactive nitrogen species (RNS) also regulate MMP activity. Oxidation of the cysteine switch within the propeptide domain can activate MMPs independently of proteolytic cleavage [16]. This mechanism is particularly relevant during inflammation and oxidative stress and may contribute to pathological alterations in MMPs function [5,16].

MMP activity is inhibited by tissue inhibitors of metalloproteinases (TIMPs), including TIMP-1, -2, -3, and -4. These proteins regulate ECM turnover and influence cell behavior, including adhesion, migration, and proliferation [17]. TIMPs bind to the active site of MMPs, thereby inhibiting their proteolytic activity. Among them, TIMP-3 is particularly important due to its role in suppressing tumor growth, inhibiting cell motility, and inducing apoptosis [17].

Dysregulation of the MMP–TIMP balance plays a critical role in various pathological conditions, including inflammation, fibrosis, abnormal angiogenesis, autoimmune diseases, and cancer [17].

## 3. MMPs in Carcinogenesis

Although tumor initiation varies among cancer types, the mechanisms underlying metastasis are largely conserved and strongly depend on MMP activity. Disruption of the balance between MMPs and TIMPs contributes to multiple stages of tumor progression [6].

In the tumor microenvironment (TME), which serves as a signaling hub, cancer cells communicate with non-cancerous cells, shifting the balance of tissue homeostasis toward disease progression [6,12]. The TME is driven by the interaction between the stroma and the epithelium. Within this environment, cancer-associated fibroblasts (CAFs) exhibit diverse functions, dividing into specialized myofibroblastic and inflammatory subtypes that transform tissues and exacerbate local inflammation [6,25].

Tumors induce profound alterations in the ECM, significantly increasing stiffness and density. Mechanical stress activates key mechanotransduction pathways, such as phosphorylation of kinases—mitogen-activated protein kinase (MAPK), extracellular signal-regulated kinase (ERK), focal adhesion kinase (FAK), and protein kinase B (Akt) [16,17]. FAK induces the tumor cell phenotype toward an invasive, migratory phenotype [7,30].

**Figure 1:**
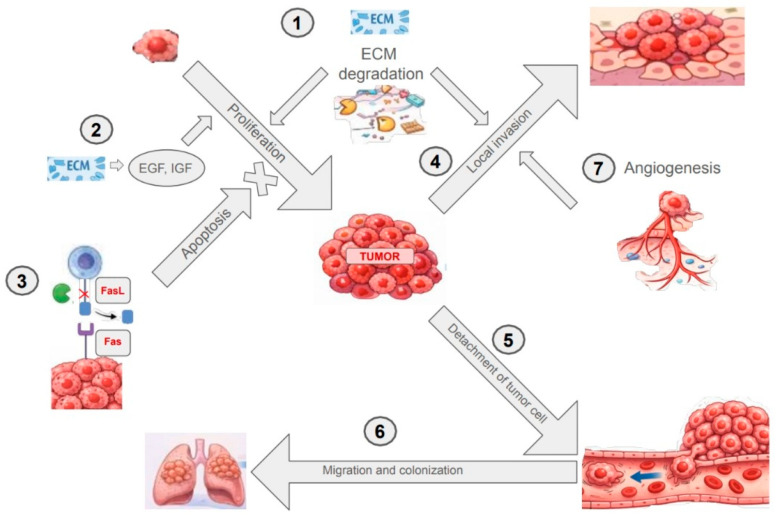
ECM Degradation (1): MMPs execute the proteolytic remodeling of the ECM (Extracellular matrix: basement membrane and interstitial stroma). Proliferation (2): MMPs modify the bioavailability of growth factors, providing sustained mitogenic divisions. Apoptotic Regulation (3): MMPs have a dual role in programmed cell death. While specific intracellular isoforms, which translocate to the nucleus, induce apoptosis, secreted isoforms play an anti-apoptotic role. They achieve this by blocking pro-apoptotic ligands (FasL), making the tumor cell resistant to apoptotic triggers. Local Invasion (4): By degrading ECM components, MMPs eliminate the physical limitations on the primary tumor, allowing for the invasion. EMT and Cellular Detachment (5): MMPs enable the Epithelial–Mesenchymal Transition (EMT) by promoting loss of cell polarity and a phenotype change. Migration (6): Released by ECM destruction fragments (matrikines), create a physical pathway that tells the cell exactly which direction to pull itself forward through the tissue. Angiogenesis (7): MMPs release pro-angiogenic factors, enabling the neovascularization.

MMPs contribute to tumor cell proliferation, local invasion, epithelial-to-mesenchymal transition (EMT), and cell detachment, migration, and colonization, apoptosis regulation, angiogenesis, and metastasis (Figure 1). Their function extends beyond only ECM degradation to include modulation of cell signaling, growth-factor activation, and regulation of cellular behavior [6].

Active matrix remodeling is also driven by infiltrating immune cells (macrophages and neutrophils) [23,31]. They are the main sources of MMP-2 and MMP-9, which remove physical barriers and open pathways for neurocellular interactions, resulting in perineural tissue invasion and neuroendocrine-induced tumor growth [32].

### 3.1. Cancer Progression and Metastasis

Metastasis is the leading cause of cancer-related mortality. It involves a multistep process including local invasion, intravasation, survival in circulation, extravasation, and colonization of distant tissues [8,9].

MMPs facilitate metastasis by degrading ECM components and basement membranes, thereby enabling tumor cell migration. They also modulate cell adhesion and signaling, influencing tumor cell interactions with the microenvironment [8].

EMT is a key process in metastasis, characterized by loss of cell polarity and acquisition of migratory properties [6]. MMPs play a critical role in EMT and are involved in both invasion and migration of cancer cells [6]. MMPs promote metastasis through three primary roles: degrading physical barriers (ECM, BM), modulating cell adhesion by modifying and forming new matrix and cell–cell attachments, breaking existing ones, and altering protein bioactivity to stimulate migration [8,9,33]. Specific MMPs involved in EMT and invasion include MMP-1, -2, -3, -7, -9, -14 [16,22,26,34].

### 3.2. Angiogenesis

Tumor growth requires the formation of new blood vessels through angiogenesis and vasculogenesis (creating vessels de novo from angioblasts) [7,10,16]. MMPs regulate these processes by remodeling the ECM and releasing pro-angiogenic factors such as VEGF and FGF-2 [6,10,11].

MMPs exhibit dual roles in angiogenesis, acting as both promoters and inhibitors depending on the context. For example, degradation of ECM components can release pro-angiogenic factors, whereas cleavage of certain substrates may generate anti-angiogenic fragments [6,17]. Authors suggest that MMP-2, -9, -14 play the main roles in tumor angiogenesis [11,30].

### 3.3. Apoptosis Regulation

Apoptosis is a natural phenomenon in the development and life of organisms, which depends on the components of the cellular apoptotic mechanisms and their quantities [31]. It is a tightly regulated process essential for maintaining tissue homeostasis. Cancer cells often evade apoptosis, contributing to uncontrolled growth [12].

MMPs influence apoptosis through multiple mechanisms. For example, MMP-7 can inhibit apoptosis by cleaving Fas ligand from the cell surface and blocking the Fas receptor stimulation, thereby disrupting apoptotic pathways (caspase-8 and caspase-10 activation) [7,10,17,27]. Conversely, MMPs may promote apoptosis through ECM degradation and disruption of cell adhesion [28].

### 3.4. Cancer Suppression

Although MMPs were viewed exclusively as tumor promoters, research shows that many exert tumor-suppressive effects. Their function is context-dependent and can simultaneously promote certain phases of cancer while restricting others [34,35,36,37].

Several MMPs (including MMP-2, MMP-7, MMP-9, MMP-12, and MMP-3) can generate angiogenesis inhibitors, such as angiostatin and endostatin, by cleaving proteins like plasminogen and type VIII collagen to effectively deprive the tumor of nutrient supply [34,38].

MMP-8 regulates the inflammatory response induced by tumors and controls cell invasion by modulating cell adhesion. It is documented as a metastasis suppressor in breast, skin, and oral cancers [34,35,36,37]. Lower levels of MMP-8 are typically associated with poor [34,35,36,37] patient outcomes [37].

MMP-12 is implicated in both tumor promotion and suppression. In lung and colon cancers, MMP-12 expression is often associated with increased patient survival due to its role in stopping tumor growth and angiogenesis [37,39].

Certain MMPs reduce cancer cell proliferation and limit distant metastasis by cleaving and modifying bioactive non-structural molecules [40].

### 3.5. Causality Challenges

In the study of matrix metalloproteinases (MMPs) in cancerogenesis, distinguishing between causality and association is a critical challenge. While elevated MMPs expression frequently correlates with advanced tumor stages, this association does not automatically mean MMPs are driving the disease; they may simply be a byproduct of the altered tumor microenvironment.

When examining this relationship, several variables must be taken into account. First, factors such as age, smoking history, comorbidities such as obesity, and medications used chronically can independently alter MMP levels.

Secondly, variability in methodology: different techniques, such as fluorimetry (which measures enzyme activity) and ELISA or immunohistochemistry (which measure protein amount), often yield conflicting results because high expression does not always equate to high functional activity. Apart from different research techniques, there are differences in the biological material used for testing—serum, plasma, or tumor tissue.

Furthermore, variations in tissue handling, storage duration, and genetic diversity across patient populations make it difficult to replicate findings. To prove causality, the field requires standardized assay protocols and rigorous validation across large, well-stratified clinical cohorts.

### 3.6. Failed Inhibitor Trials

MMPs, therefore, seemed an ideal starting point for developing anticancer therapy. Unfortunately, these studies did not yield the desired results; premature Phase II/III clinical trials on broad-spectrum MMP inhibitors, such as Marimastat and Batimastat, ended in failure [10].

MMPIs were administered to patients with advanced, end-stage metastatic disease. MMPs primarily regulate the initial stages of tissue invasion [10].

Furthermore, broad-spectrum inhibitors utilized zinc-chelating motifs (such as hydroxamates) that nonselectively inactivated entire families of MMPs, including the ADAM and ADAMTS families. This nonselective drug action resulted in the complication of musculoskeletal syndrome (MSS), which necessitated treatment discontinuation [41,42].

Blocking a single MMP often resulted in compensatory regulation of alternative pathway expression, allowing cancer cells to bypass the therapeutic blockade [42].

### 3.7. New Technologies

To overcome limitations, researchers are shifting from static abundance metrics to new functional and integrative technologies [43]. MMP activity imaging: rather than measuring total protein concentration, activity-based probes (ABPs) and zymography-based imaging are used to map active, functional enzymes directly within the tumor microenvironment in real time [43]. Functional dissection based on high-throughput CRISPR/Cas9 gene-editing platforms enables scientists to knock out or activate specific MMP genes or downstream EMT pathways [44]. This precise genetic control makes it more possible to prove causality rather than simple association by mapping direct phenotype-to-genotype consequences [44].

Genomics, transcriptomics, and proteomics allow for the accounting of clinical confounding variables by pinpointing exactly how lipid metabolism, immune signaling, and stromal interactions regulate MMP activity across diverse patient cohorts [44,45].

Combining these methods, modern oncology can finally isolate the true therapeutic vulnerabilities.

## 4. MMPs Involvement in Individual Tumors

Metalloproteinases involved in specific stages of carcinogenesis in individual cancers are presented in Table 1.

### 4.1. Breast Cancer

Breast cancer is the most common malignancy among women worldwide. Elevated expression of various MMPs has been associated with tumor progression, metastasis, and poor prognosis [46,47,48,49,50,51,52,53,54,55].

MMP-1, MMP-2, and MMP-13 have been linked to aggressive tumor behavior and reduced survival [48,49,50,51,52]. MMP-1 accumulates intracellularly during the mitotic phase and localizes to the nucleus and mitochondrial membrane, which results in resistance to apoptosis [56]. In breast cancer, the expression level of MMP-1 inversely correlates with the survival rate [5,48]. MMP-1 plays a significant role in not only primary tumor growth, but also metastasis of breast cancer to the brain, and is an unfavorable predictor of lower overall survival rate [49,50,51]. A high level of MMP-2 correlates with an unfavorable prognosis in patients with breast cancer [53]. Blocking MMP-2 secretion and activation during tumor development may contribute to decreased metastasis, and in its detection, MMP-2 may be used as a sensitive indicator [53]. MMP-13 is presented as a potential prognostic factor, as it is correlated with aggressive types of breast tumors, especially when evaluated along with Her-2/neu and lymph node status [52].

In contrast, MMP-26 may exhibit anti-tumorigenic properties in certain subtypes of breast cancer [54]. Savinov et al. [54] suggested the existence of an MMP-26-dependent pathway in patients with ERalpha/beta-positive breast cancer. Their study confirms the association between elevated MMP-26 expression in patients with ductal carcinoma in situ (DCIS) and better prognosis/survival rate [54].

The combined use of MMPs with established biomarkers, such as CA 15-3, may improve diagnostic accuracy [57,58].

### 4.2. Respiratory System

#### Lung Cancer

Lung cancer remains the leading cause of cancer-related mortality, although survival has improved in recent years [59]. MMPs act as critical regulators throughout the progression of lung cancer. MMP-1 and MMP-3 promote early tumorigenesis by inducing EMT and chronic inflammation, which remodel the tissue microenvironment to support neoplastic growth [60]. During late-stage progression, the gelatinases MMP-2 and MMP-9, along with the membrane-bound MMP-14, degrade the ECM, which allows cancer cells to intravasate into the bloodstream and migrate to distant organs [61]. Elevated MMP-9 expression is associated with poor survival and correlates with advanced tumor stage and metastasis. Interestingly, tissue overexpression of MMP-9 is linked to worse clinical outcomes [62]. MMP-9 is induced by VEGF during metastasis, and its inhibition reduces metastatic potential [63]. Among the MMPs involved in lung cancer mentioned in the literature are also MMP-7, -13, -14 [36].

### 4.3. Gastrointestinal System

#### 4.3.1. Colorectal Cancer

Colorectal cancer (CRC) is the third most common cancer and a leading cause of cancer-related mortality worldwide. MMPs play a significant role in CRC progression and metastasis.

MMP-1 has been identified as a prognostic marker in the early stages of CRC, particularly associated with tumor invasiveness and metastasis [64,65,66,67]. MMP-1 expression correlates with advanced colon cancer stage [39,65,66], and its overexpression in both primary tumors and metastatic lesions is a significant predictor of hematogenous spread to the liver and overall poor patient survival [65,67]. MMP-1 drives colorectal cancer invasion and metastasis by degrading the extracellular matrix and inducing the EMT through activation of the PI3K/Akt signaling pathway [39,66,68]. It was shown to be a key mediator of tumor self-seeding—a mechanism of primary tumor invasion, which enriches it with more aggressive cells [39]. Additionally, elevated preoperative plasma levels of MMP-1 serve as a reliable non-invasive prognostic marker for advanced disease stages and an increased risk of recurrence [64].

MMP-2 and MMP-9 are frequently overexpressed in CRC and have been proposed as potential diagnostic and prognostic biomarkers [69,70,71,72]. The literature shows that MMP-9 can demonstrate clinically relevant sensitivity and specificity for identifying colorectal cancer, especially when used in combination with other biomarkers (CEA, TIMP-1) or in fecal testing [73,74,75,76].

MMP-7 has also been associated with poor prognosis and may serve as an independent prognostic factor in advanced CRC [77,78,79,80].

Overall, MMP-based biomarkers show promise but require further validation.

#### 4.3.2. Gastric Cancer

Gastric cancer remains a major global health challenge. MMPs, particularly MMP-2, MMP-7, and MMP-9, are involved in tumor progression, angiogenesis, and metastasis.

MMP-7 was found to be upregulated in epithelium in the case of H. pylori-associated gastritis, which is a risk factor for GC [81].

MMP-9 expression is significantly increased in poorly differentiated gastric carcinomas, tumors with serosal perforation, and TNM stage III–IV tumors, which assigns a significant role for MMP-9 in the diagnosis and specification of primary tumor invasion, grade, and TNM stage in gastric carcinoma [82]. Mroczko et al. [83] confirmed in their study that MMP-9 is produced not only by cancer cells but also by the inflammatory infiltrating cells present in gastric tumors. Moreover, they identified a correlation between MMP-9 immunoreactivity and several clinicopathological characteristics of gastric cancer, including TNM stage, tumor invasion depth, and the presence of lymph node or distant metastases [83].

Overexpression of the above enzymes has been associated with poor prognosis and may have potential as diagnostic and prognostic biomarkers [81,82,83,84,85,86,87].

#### 4.3.3. Pancreatic Cancer (PDAC)

Pancreatic ductal adenocarcinoma (PDAC) accounts for over 90% of pancreatic cancers and is characterized by a very poor prognosis, with a 5-year survival rate of approximately 9% [59,88]. Dysregulation of matrix metalloproteinases (MMPs) is commonly observed in PDAC [88,89]. MMP-2, MMP-9, and MMP-7 are most strongly implicated in disease development; their activity correlates with the tumor’s ability to degrade the peritumoral tissue and facilitate systemic dissemination [90]. High MMP-2 expression is associated with higher histological grade and poor prognosis [91]. Increased levels of gelatinases MMP-2 and MMP-9 are associated with tumor progression and angiogenesis [92,93]. Moreover, expression of MMP-2, MMP-7, and MMP-9 correlates with histopathological features such as inflammation, necrosis, and neovascularization [94]. MMP-7 remodels the ECM and activates oncogenic signaling pathways by releasing growth factors and triggering EMT [95]. It initiates pancreatic carcinogenesis by driving Acinar-to-Ductal Metaplasia (ADM), a process where healthy acinar cells transform into ductal cancer precursors, and creates a dense, hypoxic microenvironment that promotes tumor cell survival [95].

### 4.4. Genitourinary System

#### 4.4.1. Prostate Cancer

Prostate cancer is one of the most common malignancies in men and a leading cause of cancer-related mortality [96]. Dysregulation of MMP homeostasis plays a significant role in its progression. Increased expression of MMP-2 and MMP-9 has been associated with higher Gleason score, advanced stage, and poor prognosis [97,98,99]. It was reported that MMP-14 is a key regulator of metastatic potential through activation of MMP-2 and promotion of cell migration [100]. MMP-7 and MMP-9 also play an important role in the metastasis of tumor cells [99,101,102]. MMP-7 promotes bone metastasis by cleaving RANKL, a protein that activates osteoclasts. This process leads to rapid bone resorption, creating space for the tumor to expand and releasing growth factors from the bone matrix [103]. Within the bone microenvironment, MMP-9 processes Vascular Endothelial Growth Factor (VEGF-A) into a highly bioavailable form, triggering angiogenesis [103].

MMPs represent important contributors to prostate cancer development and may serve as potential diagnostic and prognostic biomarkers as well as therapeutic targets.

#### 4.4.2. Ovarian Cancer

Despite advances in therapies, ovarian cancer has the highest mortality among gynecological malignancies, largely due to late diagnosis [104]. MMPs (2, 3, 9, and 14) are involved in all stages of disease progression, including EMT, ECM degradation, and angiogenesis [105]. MMP-14 is a key regulator of metastasis [106], while MMP-2 and MMP-9 promote cell motility and matrix remodeling [107,108,109]. Among the tested MMPs, MMP-1, MMP-7, MMP-26, and MMP-10 show the highest diagnostic potential as a possible prognostic biomarker [110,111]. However, they play a different role in the development of ovarian cancer. High expression of MMP-8, MMP-9, and MMP-14 is associated with poor prognosis, whereas MMP-7 and MMP-25 may have protective roles [112]. High MMP-9 levels correlate with the presence of metastatic foci and the formation of “vasculogenic-like networks” (vascular mimicry), which provide alternative pathways for cancer cell spread [112]. MMP-14 is typically involved in the activation of other MMPs (like MMP-2) and in the direct degradation of the ECM, facilitating the invasion of cancer cells into surrounding tissues [112]. The protective role of MMP-7 contrasts with its known pro-invasive role in other cancers [112]. Overall, multiple MMPs have potential utility in the diagnosis, monitoring, and prognosis of ovarian cancer.

### 4.5. Skin

#### Melanoma

Melanoma accounts for less than 5% of skin cancers but is the leading cause of skin cancer-related death due to its metastatic potential [113,114]. MMPs act as primary drivers of the transition from radial to vertical growth phases and are critical for metastatic dissemination. It was described that MMP-1, -2, -3, -9, -13, and -14 contribute to melanoma progression [115].

Upregulated expression of MMP-2 is critical for the remodeling of the tumor microenvironment and facilitates the transition from localized melanocytic lesions to highly invasive phenotypes [113].

Increased MMP-9 expression is associated with greater tumor aggressiveness, reduced overall survival, and may predict distant metastasis [113,115,116,117,118].

### 4.6. Nervous System

#### Glioma

Gliomas are the most common central nervous system tumors, with glioblastoma (grade IV) characterized by high aggressiveness and poor prognosis [119]. Increased serum levels of MMP-2 and MMP-9 are associated with malignancy and poor outcomes. Their expression correlates with tumor recurrence, with higher levels observed in recurrent versus primary gliomas [120,121]. Overexpression of MMP-2 and MMP-9 is typical of malignant brain tumors, indicating their potential as diagnostic, prognostic, and therapeutic targets [119,120,122,123,124,125,126,127].

### 4.7. Endocrine System

#### 4.7.1. Thyroid Cancer

Thyroid cancer is the most common endocrine malignancy [128]. Increased expression of MMPs, particularly MMP-1, MMP-2, and MMP-9, has been associated with tumor progression, invasion, and metastasis.

MMP-1 acts as a primary regulator of tumor dedifferentiation, facilitating the lethal transition from well-differentiated papillary thyroid carcinoma (PTC) to aggressive poorly differentiated or ATC [129].

MMP-2 has shown potential as a diagnostic and prognostic biomarker in PTC, with correlations observed between serum levels and tumor aggressiveness [130,131]. High preoperative serum levels of MMP-2 serve as a precise diagnostic tool to differentiate PTC from benign thyroid nodules and correlate with increased risk of central lymph node involvement [132,133].

MMP-9 has been implicated in ECM degradation, angiogenesis, EMT, and tumor invasiveness, although its diagnostic utility remains controversial [134,135]. Li et al. [136] highlight that MMP-9 impact varies across different types of thyroid carcinoma: high MMP-9 expression is linked to more aggressive behavior and recurrence in PTC, and in ATC, MMP-9 levels are often extremely high, contributing to rapid local invasion and a poor prognosis.

#### 4.7.2. Parathyroid Gland

Primary hyperparathyroidism is most commonly caused by a single adenoma. The role of MMPs in parathyroid pathology remains poorly understood.

Limited studies suggest a potential involvement of MMP-2 and MMP-9 in tumor biology and the inflammatory processes associated with the disease [137,138]. Elevated MMP-9 in patients with hyperparathyroidism reflects a state of low-grade systemic inflammation and active ECM remodeling [137]. Further research is required to clarify their diagnostic and therapeutic relevance.

**Table 1:** Metalloproteinases involved in specific stages of carcinogenesis. In vivo studies are marked with an asterisk.

Process	MMPs Involved	Representative Cancers
ECM degradation	MMP-1	Breast cancer [46], Lung cancer [60], PDAC [139], Gastric cancer [140], Ovarian Cancer [141], Melanoma [115,142], Thyroid cancer [129]
	MMP-2	Breast cancer [46,143,144,145], Lung cancer [60], PDAC [139], Gastric cancer [140], Ovarian cancer [146], Melanoma [118], Glioma [147], Thyroid cancer [130]
	MMP-3	Breast cancer [46], Prostate cancer [148]
	MMP-7	PDAC [139], Gastric cancer [140]
	MMP-9	Breast Cancer [143,144], Lung cancer [60], Colorectal cancer [149], PDAC [139], Gastric cancer [140], Ovarian cancer [146], Melanoma [118], Glioma [147], Thyroid cancer [134,135] *, Parathyroid tumor [137]
	MMP-13	Breast Cancer [150], Lung cancer [60], PDAC [139], Gastric cancer [140,145], Ovarian cancer [141]
	MMP-14	Lung cancer [60], PDAC [139], Gastric cancer [140], Glioma [6,147]
Tumor proliferation/growth	MMP-1	Breast cancer [49,50,51] *, Colorectal cancer [151], Thyroid cancer [129]
	MMP-2	Lung cancer [60], PDAC [152], Gastric cancer [153], Prostate cancer [100]
	MMP-3	Prostate cancer [100], Ovarian cancer [105]
	MMP-7	Lung cancer [60], Colorectal cancer [151], PDAC [152], Gastric cancer [153]
	MMP-9	Lung cancer [60], PDAC [152], Gastric cancer [153], Prostate cancer [100], Ovarian cancer [105], Thyroid cancer [136]
	MMP-14	Lung cancer [60], PDAC [152], Gastric cancer [153], Prostate cancer [100]
Apoptosis regulation	MMP-1	Breast cancer [56]
	MMP-2	Lung cancer [60], PDAC [152], Gastric cancer [153], Ovarian cancer [154], Melanoma [155]
	MMP-3	Prostate cancer [100]
	MMP-7	Colorectal cancer [77,78,79,80], Gastric cancer [153], PDAC [152], Prostate cancer [100], Glioma [156,157]
	MMP-9	Lung cancer [60], PDAC [152], Gastric cancer [153], Prostate cancer [100], Thyroid cancer [136], Melanoma [155]
	MMP-11	Prostate cancer [100]
	MMP-14	Lung cancer [60], PDAC [152], Gastric cancer [153]
	MMP-15	Prostate cancer [100]
Epithelial–mesenchymal transition (EMT) and EMT-like transition	MMP-2	Lung cancer [60], Colorectal cancer [149], Gastric Cancer [158]
	MMP-3	Breast cancer [159], Prostate cancer [100], Ovarian cancer [105]
	MMP-7	Gastric cancer [145,158], Prostate cancer [100]
	MMP-9	Breast cancer [149], Lung cancer [60], Gastric cancer [158], Prostate cancer [100], Ovarian cancer [105], Glioma [160], Thyroid cancer [134,135,136] *
	MMP-11	Prostate cancer [100]
	MMP-14	Lung cancer [60], Gastric cancer [158], Ovarian cancer [105], Glioma [160]
	MMP-28	Lung cancer [145]
Metastasis	MMP-1	Breast cancer [49,50,51] *, Lung cancer [60], Colorectal cancer [151], PDAC [139], Gastric cancer [161], Prostate cancer [100], Thyroid cancer [129]
	MMP-2	Breast cancer [46,53,162], Lung cancer [60], Colorectal cancer [151], PDAC [139], Gastric cancer [161], Ovarian cancer [105], Prostate cancer [100]. Thyroid cancer [134,135,136] *
	MMP-3	Prostate cancer [100]
	MMP-7	Lung cancer [60], Colorectal cancer [151], PDAC [139], Gastric cancer [161], Prostate cancer [100]
	MMP-9	Breast cancer [46], Lung cancer [60], Colorectal [151], PDAC [139], Gastric cancer [161], Ovarian cancer [105], Prostate cancer [100], Melanoma [163], Thyroid cancer [136]
	MMP-10	Ovarian cancer [164]
	MMP-11	Colorectal cancer [151], Ovarian cancer [164]
	MMP-13	Lung cancer [60], PDAC [139], Gastric cancer [161], Prostate cancer [100], Glioma [147]
	MMP-14	Lung cancer [60], PDAC [139], Gastric cancer [161], Ovarian cancer [105], Prostate cancer [100]
	MMP-16	Glioma [147]
	MMP-26	Prostate cancer [100]
Angiogenesis	MMP-1	Breast cancer [159], Prostate cancer [100], Glioma [165]
	MMP-2	Lung cancer [60,149], PDAC [93,166], Gastric cancer [167], Ovarian cancer [34,105], Prostate cancer [100], Glioma [156]
	MMP-7	PDAC [145], Prostate cancer [100]
	MMP-9	Lung cancer [60], PDAC [93,166], Gastric cancer [167], Ovarian cancer [34,105]. Prostate cancer [100], Glioma [149,156]
	MMP-14	Breast cancer [145], Lung cancer [60], Colorectal cancer [151], PDAC [166], Gastric cancer [167], Ovarian cancer [34,105], Prostate cancer [100]

## 5. Conclusions

The role of MMPs in cancer development and progression is complex and context-dependent. While many MMPs promote tumor growth, invasion, and metastasis, others may exert protective effects.

Current evidence indicates that non-selective inhibition of all MMPs is unlikely to be an effective therapeutic strategy. Instead, a more targeted approach focusing on specific MMPs and disease stages may be required.

Further research is essential to better understand biology and to develop selective therapeutic strategies and reliable biomarkers for cancer diagnosis and treatment.

## Data Availability

The original contributions presented in this study are included in the article. Further inquiries can be directed to the corresponding author.
